# Chronic Exercise Training Improved Aortic Endothelial and Mitochondrial Function via an AMPKα2-Dependent Manner

**DOI:** 10.3389/fphys.2016.00631

**Published:** 2016-12-21

**Authors:** Xiaohui Chen, Xiangbo An, Dongrui Chen, Maoqing Ye, Weili Shen, Weiqing Han, Youyi Zhang, Pingjin Gao

**Affiliations:** ^1^Laboratory of Vascular Biology and Key Laboratory of Stem Cell Biology, Institute of Health Sciences, Shanghai Institutes for Biological Sciences, Chinese Academy of Sciences, University of Chinese Academy of SciencesShanghai, China; ^2^Institute of Vascular Medicine, Peking University Third HospitalBeijing, China; ^3^Shanghai Key Laboratory of Hypertension, Ruijin Hospital, Shanghai Jiao Tong University School of MedicineShanghai, China; ^4^Shanghai Institute of HypertensionShanghai, China

**Keywords:** AMPK, exercise, vascular, endothelial function, mitochondrial function

## Abstract

Chronic exercise training is known to protect the vasculature; however, the underlying mechanisms remain obscure. The present study hypothesized that exercise may improve aortic endothelial and mitochondrial function through an adenosine monophosphate-activated protein kinase α2 (AMPKα2)-dependent manner. Ten-week-old AMPKα2 knockout (AMPKα2^−/−^) mice and age-matched wild-type (WT) mice were subjected to daily treadmill running for 6 weeks, and the thoracic aorta from these mice were used for further examination. Our results showed that exercise significantly promoted vasodilatation and increased expression and phosphorylation of endothelial nitric oxide synthase (eNOS), concomitant with increased AMPKα2 expression in WT mice. These effects were not observed in AMPKα2^−/−^ mice. Furthermore, exercise training increased thoracic aortic mitochondrial content as indicated by increased Complex I and mitochondrial DNA (mtDNA) in WT mice but not in AMPKα2^−/−^ mice. This may be caused by decreased mitochondrial autophagy since the expression of BH3 domain-containing BCL2 family members BNIP3-like (BNIP3L) and LC3B were decreased in WT mice with exercise. And these changes were absent with AMPKα2 deletion in mice. Importantly, exercise increased the expression of manganous superoxide dismutase (MnSOD) and catalase, suggesting that mitochondrial antioxidative capacity was increased. Notably, the improved antioxidative capacity was lost in AMPKα2^−/−^ mice with exercise. In conclusion, this study illustrated that AMPKα2 plays a critical role in exercise-related vascular protection via increasing endothelial and mitochondrial function in the artery.

## Introduction

It is well documented that exercise training can effectively prevent cardiovascular risk factors such as obesity, hypertension, and diabetes in the long term (Stewart, 2002). For example, it has been shown that exercise improved vascular endothelial function in hypertensive animal models (Kumral et al., 2016) and patients with coronary artery disease (Hambrecht et al., 2000). In vessel samples from animals with exercise, the expression and Ser^1177^ phosphorylation of endothelial nitric oxide synthase (eNOS) were increased, whereas the oxidative stress was decreased. These changes lead to increased NO availability and improved vascular function (Kojda et al., 2001; Hambrecht et al., 2003; Adams et al., 2005). However, the mechanisms by which exercise exerts these beneficial effects on the vasculature are little known.

Adenosine monophosphate-activated protein kinase (AMPK) is a serine/threonine kinase consisting of α, β, and γ subunits. The β and γ regulatory subunits maintain the stability of the kinase, and the α subunit possesses catalytic activity (Steinberg and Kemp, 2009). In particular, two isoforms of AMPKα, AMPKα1, and AMPKα2 are both expressed in endothelial cells and in smooth muscle cells (Goirand et al., 2007). Furthermore, it has been shown that AMPKα2 is increased during exercise in skeletal muscle cells (Magnoni et al., 2014), and the increased AMPKα2 can regulate gene and protein expression through direct interaction with the nucleus (McGee et al., 2003; Jørgensen et al., 2006). Recent studies suggest that AMPK has a much more important role in the vasculature as it activates and phosphorylates endothelial nitric oxide synthase (eNOS) (Morrow et al., 2003), protects endothelial cells against oxidative stress (Schulz et al., 2008) and prevents vascular smooth muscle proliferation (Nagata et al., 2004). These results may suggest a protective role of AMPK in the vascular system.

Mitochondria are mobile organelles that exist in dynamic networks. To maintain a healthy population of mitochondria, the content of mitochondria is critically regulated by biogenesis, fusion-fission, and autophagy. These regulations ultimately determine the quantity, quality, and function of mitochondria, thereby contributing to cell function (Yan et al., 2012). It has been shown that maintaining mitochondrial content and functional network is crucially important for proper function of both endothelial cells and vascular smooth muscle cells (Salabei and Hill, 2013; Szewczyk et al., 2015). As expected, the abnormal content and network regulation of mitochondria lead to various cardiovascular diseases, such as diabetic vascular dysfunction (Pangare and Makino, 2012) and hypertension (Jin et al., 2011). It has been shown that exercise can increase mitochondrial content and function in skeletal muscle (Russell et al., 2014). In addition, mitochondrial antioxidant enzymes can reduce the damaging effects of reactive oxygen species (ROS, Tang et al., 2014). For example, mitochondrial manganese superoxide dismutase (MnSOD) deficiency aggravated age-dependent vascular dysfunction and increased mitochondrial oxidative stress (Wenzel et al., 2008). Over-expressing mitochondria MnSOD in mice attenuated angiotensin II (Ang-II) induced hypertension (Dikalova et al., 2010). However, whether exercise training could improve mitochondrial function in the vasculature remains unclear. In the present study, we hypothesize that chronic exercise training may improve endothelial function and mitochondrial function in aortas and that AMPKα2 may contribute these protective effects by mediating the expression of the corresponding proteins.

## Materials and methods

### Animals and exercise protocol

All animal treatment complied with the *Guide for the Care and Use of Laboratory Animals* published by the US National Institutes of Health (NIH Publication No. 85–23, revised 1996). All animal procedures were approved in accordance with the institutional guidelines established by the *Committee of Ethics on Animal Experiments at the Chinese Academy of Sciences*. Wild type (WT) mice were provided by the Institute of Laboratory Animal Science of Peking Union Medical College. AMPKα2-knockout (AMPKα2^−/−^) mice were kindly provided by Dr. Benoit Viollet (Department of Endocrinology, Metabolism and Cancer, Institute Cochin, University Paris Descartes, Paris, France) and bred in a specific pathogen-free environment. Male AMPKα2^−/−^ and WT mice were both with C57BL/6J genetic background. All mice were 2 months old with a mean body weight of 18 ± 2 g at the start of the experiment.

After allowing acclimatization to their housing and the treadmill, WT mice (*n* = 20), and AMPKα2^−/−^ mice (*n* = 20) were randomly divided into two groups: the control group and the training group, with 10 mice in each group. Mice in the training group ran on the treadmill for 90 min/day at 9.0 meters/min (0% grade), 5 days/week for 6 weeks (Fernando et al., 1993). Body weight, heart rate and systolic/diastolic blood pressure were assessed in all animals. After 12 h of the last training, mice were anesthetized of pentobarbital (5 mg/100 g) with an intraperitoneal injection and sacrificed.

### Western blot analysis

The thoracic aortas were dissected out and immersed in liquid nitrogen immediately. Then the frozen tissues were lysed in RIPA (Radio Immunoprecipitation Assay) buffer containing 150 mM NaCl, 50 mM Tris (pH 7.4), 1% sodium deoxycholate, 1% Triton X-10, 0.1% SDS, protease inhibitor (sodium fluoride, sodium orthovanadate, leupeptin, EDTA) (Beyotime, Haimen, China). After sonication on ice for 30 min and centrifugation at 12 000 rpm for 20 min at 4°C, the supernatant was collected for Western blotting as previously described (Li et al., 2012). The primary antibodies were as follows: anti-MnSOD (ABclonal, MA, USA), anti-AMPKα2 (Abcam, Cambridge, England), anti-phospho-AMPKα1/α2 (Thr^172^), anti-BNIP3L (BH3 domain-containing BCL2 family members BNIP3-like) (Bioworld, St. Louis, Park, USA), anti-eNOS, anti-phospho-eNOS (Ser^1177^) (BD Biosciences, NJ, UK), anti-Complex I, anti-PGC-1α (peroxisome proliferator-activated receptor gamma coactivator 1 alpha), anti-Drp1(dynamin related protein 1), anti-Mfn1 (mitofusin 1), anti-LC3, anti-catalase, anti-GAPDH (Santa Cruz, CA, USA), anti-mTOR (mammalian target of rapamycin), anti-phospho-mTOR (Ser^2448^) (Sigma-Aldrich, St. Louis, MO, USA). Immunoreactive bands were highlighted by electrochemiluminescence (ECL) technology and quantified by densitometry using imaging software (Image Jversion 1.46, NIH, Maryland, USA). The individual values were originally expressed as a percentage of a target protein and an internal protein standard (GAPDH) (target protein content/GAPDH content) and then expressed as a fold change of the normal WT control group (target protein content/GAPDH content) value.

### Immunofluorescence

The paraffin sections were deparaffinized by dimethylbenzene and rehydrated by graded alcohol. Antigen retrieval was processed by citric acid buffer (pH 6.0) for 5 min at 100°C. Then the slides were incubated in hydrogen peroxide for 10 min and were blocked in TBST (tris-buffered saline and tween) containing 5% Bovine Serum Albumin at room temperature for 30 min. Some sections were subsequently incubated with 300 nM MitoTracker Green (Invitrogen, CA, USA) at room temperature for 30 min. Other sections were incubated at 4°C overnight with antibodies against AMPKα2 (1:100, Abcam, Cambridge, England), fluorescent anti-rabbit secondary antibody at a 1:400 dilution for 30 min, and then nucleus dye 4,6-diamidino-2-phenylindole (DAPI) for 3 min. All images were taken by using a Zeiss Pascal LSM 710 confocal microscope (Germany). Fluorescence intensity was analyzed with Image Pro Plus in three independent samples.

### Mitochondrial DNA copy number

Genomic DNA of the thoracic aorta tissue was extracted by using UniversalGen DNA Kit(Cwbiotech, Beijing, China). The mitochondrial (mt) copy number was analyzed by real-time PCR (ABI 7900 Real Time PCR System; Foster City, CA) as previously described (Ray Hamidie et al., 2015), through the relative value of mitochondrial and nuclear DNA (mt:nuclear DNA) which reflects the amounts of mitochondria per cell. The mitochondrial DNA (mtDNA) forward primer was CCTAGGGATAACAGCGCAAT (5′-3′) and the reverse primer was ATCGTTGAACAAACGAACCA. The nuclear DNA (nDNA) forward primer was AGAGCTCTGCGGGTACATCT and the reverse primer was CATCAGTGACGGTGCCTTAC. Q-PCR were performed in a real time PCR system: the PCR began with 95°C denaturation for 30 s followed by 40 cycles of 95°C denaturation for 5 s, and annealing and elongation for 34 s at 60°C. Samples were assayed in triplicate. Cycle threshold (CT) was used for data analysis, and CT (nDNA)—CT (mtDNA) or ΔCT was used to reflect the difference in CT values. Results were expressed as the copy number of mtDNA per cell, 2 × 2^−ΔCT^.

### Thoracic aorta ring assay

Mice thoracic aortas were separated, cleared of fat and connective tissues, cut into 2–3 mm rings, and fixed on isometric force transducers (Danish Myo Technology Model 610 M, Denmark) in a 5 ml organ bath, and aerated with 95% O_2_ and 5% CO_2_ under an initial resting tension of 2.5 mN (Zhou et al., 2014). Force was recorded in a PowerLab/8sp data acquisition system (A.D. Instruments, Castle Hill, Australia). After 1 h of incubation in oxygenated Krebs' medium (containing: KCl 4.7 mmol/L, NaCl 118 mmol/L, CaCl_2_ 2.5 mmol/L, KH_2_PO_4_ 1.2 mmol/L, MgSO_4_ 1.2 mmol/L, glucose 11 mmol/L and NaHCO_3_ 25 mmol/L) at pH 7.4 and 37°C, rings contractility was tested 3 times by high K^+^ mediums (60 mM KCI) to stabilize the contraction. Cumulative response curve of phenylephrine (10^−8^ to 10^−4^ mol/L) was performed to assess the vasoconstriction response and cumulative concentration-response curves of acetylcholine (10^−8^ to 10^−4^ mol/L) were constructed with a phenylephrine pre-contraction (3 μmol/L).

### Statistical analysis

All values are reported as means ± SD. Comparison of groups involved Student's unpaired two tailed *t*-test or two-way ANOVA with the Bonferroni test for post-hoc analysis (SigmaPlot Software, San Jose, CA, USA). *P* < 0.05 was considered statistically significant.

## Results

### The protein expression and phosphorylation of AMPKα2 were increased in mice aorta with chronic exercise

Firstly, there was no significant difference of body weight and systolic blood pressure in WT and AMPKα2^−/−^ mice pre and post exercise as shown in Table S1. Table S1 also showed that heart rate was decreased in the exercise group by 17.7%, which was comparable in WT and AMPKα2^−/−^ mice.

**Figure 1:**
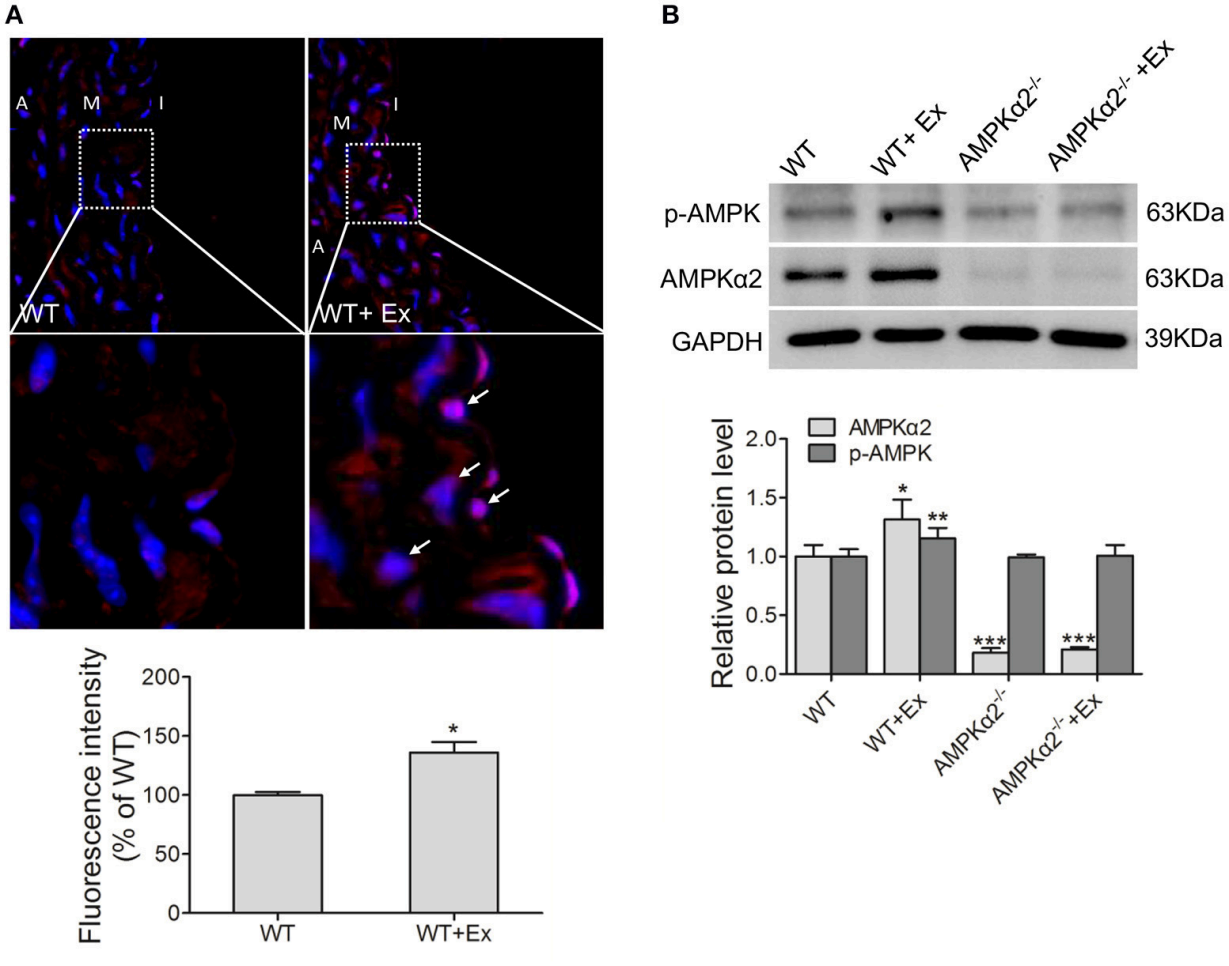
**Exercise training increased thoracic aortic AMPKα2 activity. (A)** Representative immunofluorescence images and fluorescence intensity analysis showing the expression of AMPKα2 in aorta, in which fluorescence-positive cells were stained in red (arrow) and nuclei were counterstained with DAPI (blue). **(B)** Representative western blot images and summarized data showing the expression of p-AMPKα2 and AMPKα2 of aorta from WT and AMPKα2 knockout mice with or without exercise. The protein levels of p-AMPKα2 and AMPKα2 were normalized to GAPDH. WT, wild type; Ex, exercise. Values are mean ± SD (*n* = 6 in each group). ^*^p < 0.05; ^**^p < 0.01 versus WT. ^***^p < 0.001 vs. WT+Ex. I indicates intima; M, media; A, adventitia.

Next, we evaluated whether chronic exercise would have any effect on AMPKα2 expression. As shown in Figure 1A, AMPKα2 expression was dramatically increased in the aorta after chronic exercise training in immunofluorescence staining, although the overall vascular architecture had no significant difference in the four groups of mice in H&E staining (Figure S1). This was consistent with western blot results showing that exercise training induced a significant increase in aortic AMPKα2 protein expression by 31% in WT mice (Figure 1B). Furthermore, it was found that phosphor-AMPKα (p-AMPKα) (T172) was also significantly increased in the aorta of exercised mice by using phosphor-specific antibody against both α1 and α2 isoforms of AMPK. In AMPKα2^−/−^ mice, the protein of AMPKα2 was not detectable due to gene knockout, and p-AMPKα (T172) had similar basal levels to WT mice but showed no increase in response to exercise. These results indicated that exercise induced a significant increase in the expression and phosphorylaton of AMPKα2 in the aorta.

### AMPKα2 deficiency decreased vasodilation and eNOS of aorta in exercise

**Figure 2:**
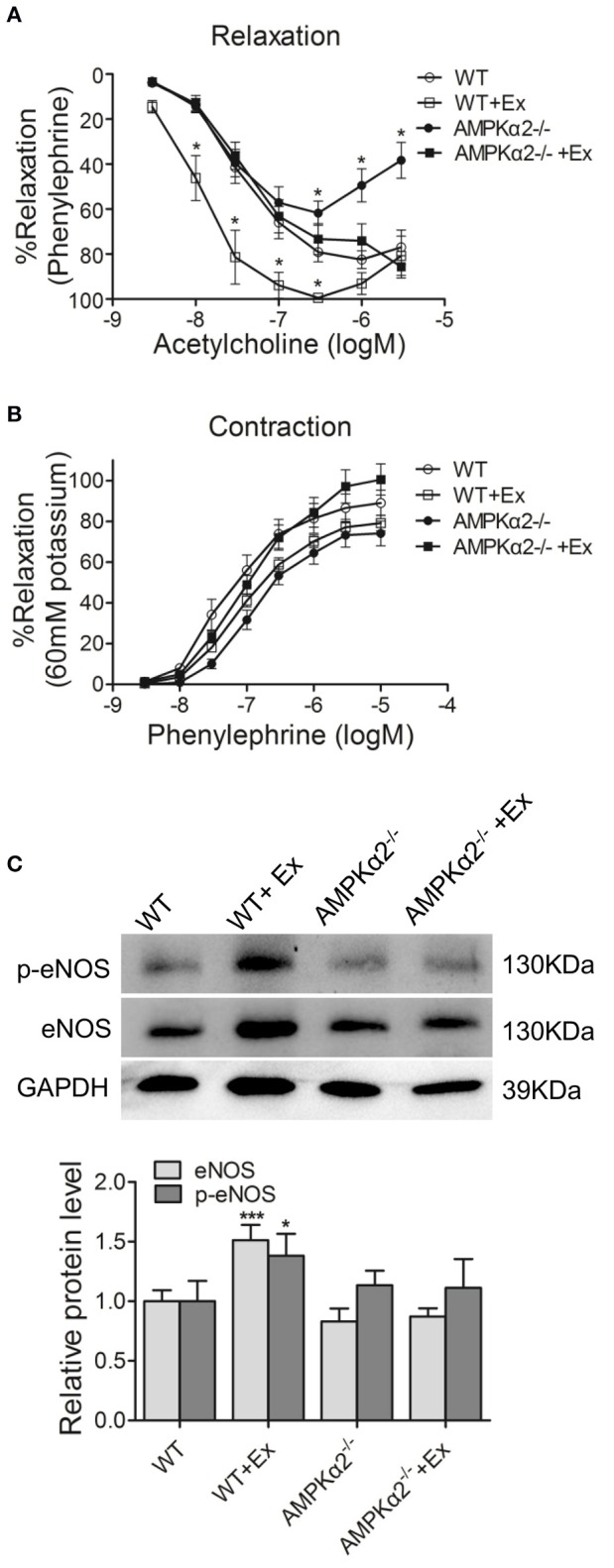
**Exercise improved vasodilation and endothelial function in an AMPKα2-dependent manner. (A)** Dose-dependent vasorelaxation to acetylcholine of aorta from WT and AMPKα2 knockout mice with or without exercise. **(B)** Dose-dependent vasocontraction to phenylephrine of aorta from WT and AMPKα2 knockout mice with or without exercise. **(C)** Representative western blot images and summarized data showing the expression of p-eNOS and eNOS of aorta from WT and AMPKα2 knockout mice with or without exercise. The protein levels of p-eNOS and eNOS were normalized to GAPDH. WT, wild type; Ex, exercise. Values are mean ± SD (*n* = 6 in each group). ^*^p < 0.05; ^***^p < 0.001 versus WT.

We then analyzed whether exercise would have any beneficial effect on vasodilation. As shown in Figure 2A, the vascular relaxation to acetylcholine was decreased in aorta rings from AMPKα2^−/−^ mice compared with age-matched wild type mice, indicating that AMPKα2 was involved in NO-dependent vasodilation. Importantly, the improvement of vasodilation was significantly lower in AMPKα2^−/−^ mice compared with WT mice, although exercise increased the vasorelaxation ability of the aorta in both WT and AMPKα2^−/−^ mice (Figure 2A). These results indicated that AMPKα2 played an important role in exercise-related vasorelaxation. In contrast, the vasoconstriction of aortas responding to phenylephrine was similar among the four groups (Figure 2B), suggesting that exercise might have no effect on vasocontraction.

We then further investigated whether the difference in vasodilation was due to changes in eNOS/p-eNOS and the possible involvement of AMPKα2. As expected, WT mice with exercise exhibited increased eNOS protein expression and phosphorylation in aorta compared with WT mice without exercise (Figure 2C). In contrast, AMPKα2^−/−^ mice with exercise did not show any increase in total eNOS level or p-eNOS level in aorta compared with AMPKα2^−/−^ mice without exercise. These results indicated that the improved vasodilation of aortas during exercise training in mice might be through an AMPKα2-dependent mechanism.

### AMPKα2 deficiency results in loss of aortic mitochondrial content increase with exercise

Accumulating studies indicated that mitochondrial content plays a critical role in maintaining vascular function. We thus evaluated whether exercise would have any effect on aortic mitochondrial content and the possible involvement of AMPKα2 by assessing mitochondrial fluorescence intensity, mtDNA copy number and Complex I protein expression.

**Figure 3:**
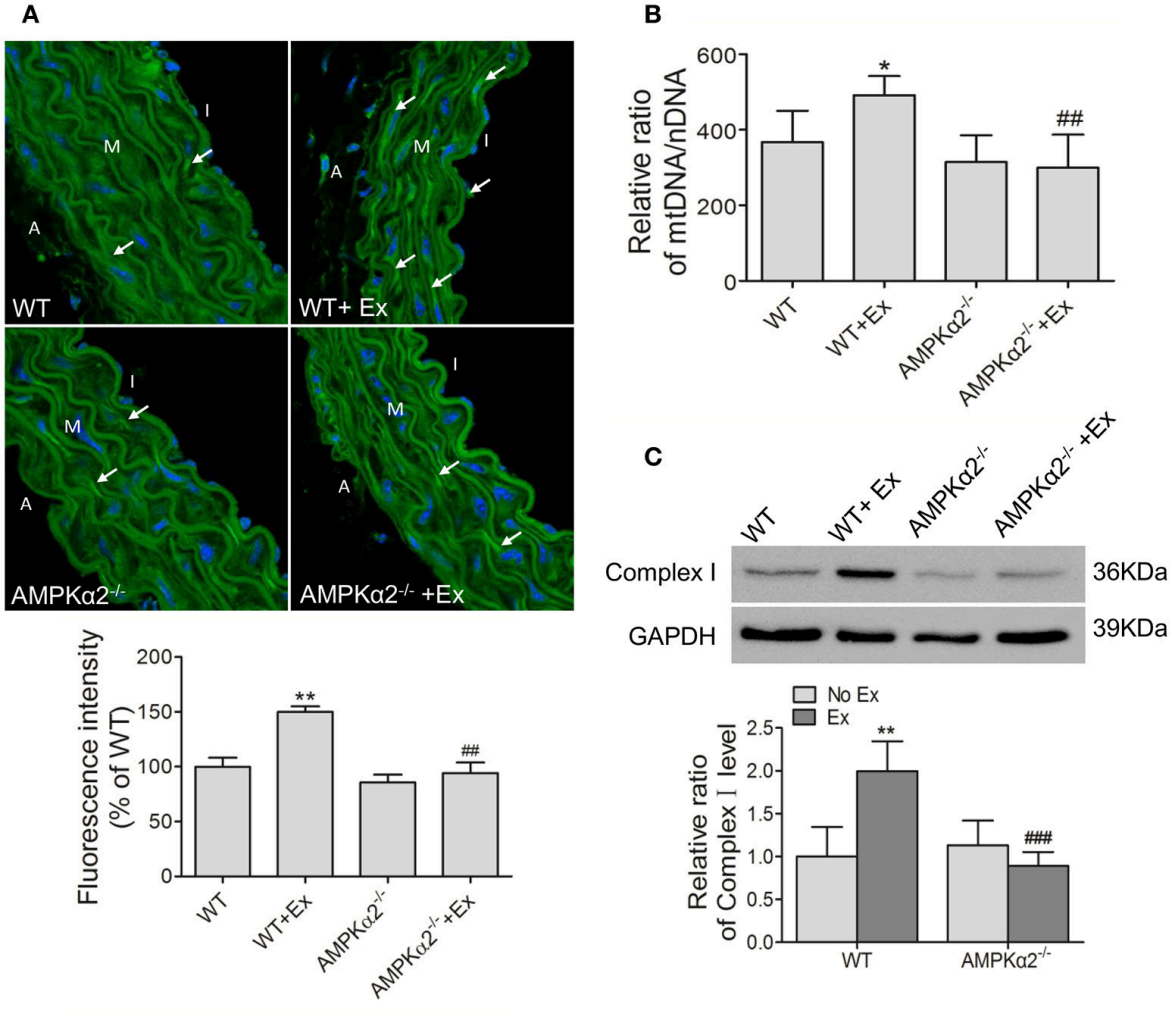
**Exercise training increased aortic mitochondrial content connected with AMPKα2. (A)** Representative MitoTracker Green immunofluorescent images and fluorescence intensity analysis of aorta from WT and AMPKα2 knockout mice with or without exercise. Mitochondria were stained in green (arrow) and nuclei were counterstained with DAPI (blue). **(B)** Summarized data showing the mitochondria DNA copy number of aorta from WT and AMPKα2 knockout mice with or without exercise. **(C)** Representative western blot images and summarized data showing the expression of Complex I of aorta from WT and AMPKα2 knockout mice with or without exercise. The mitochondria DNA copy numbers were normalized to nuclear DNA, and the protein levels of Complex I were normalized to GAPDH. WT, wild type; Ex, exercise. Values are mean ± SD (*n* = 6 in each group). ^*^p < 0.05; ^**^p < 0.01 versus WT; ^*##*^*p* < 0.01; ^*###*^*p* < 0.001 versus WT+Ex. I indicates intima; M, media; A, adventitia.

As shown in Figure 3A, MitoTracker Green fluorescence intensity increased significantly in WT mice with exercise compared with WT mice without exercise. Consistently, the mtDNA copy number was upregulated by 34% in WT mice during exercise (Figure 3B). Meanwhile, Complex I protein expression also showed an increase of 2-folds in WT exercise mice compared to control mice (Figure 3C). In contrast, AMPKα2^−/−^ mice with exercise did not show any increase in mitochondrial fluorescence intensity, mtDNA copy number and Complex I protein compared with AMPKα2^−/−^ mice without exercise (Figure 3). These data suggested that exercise increased aortic mitochondrial content, and this effect was dependent on the presence of AMPKα2.

### Effect of AMPKα2 deficiency on autophagy of aortic mitochondria with exercise

**Figure 4:**
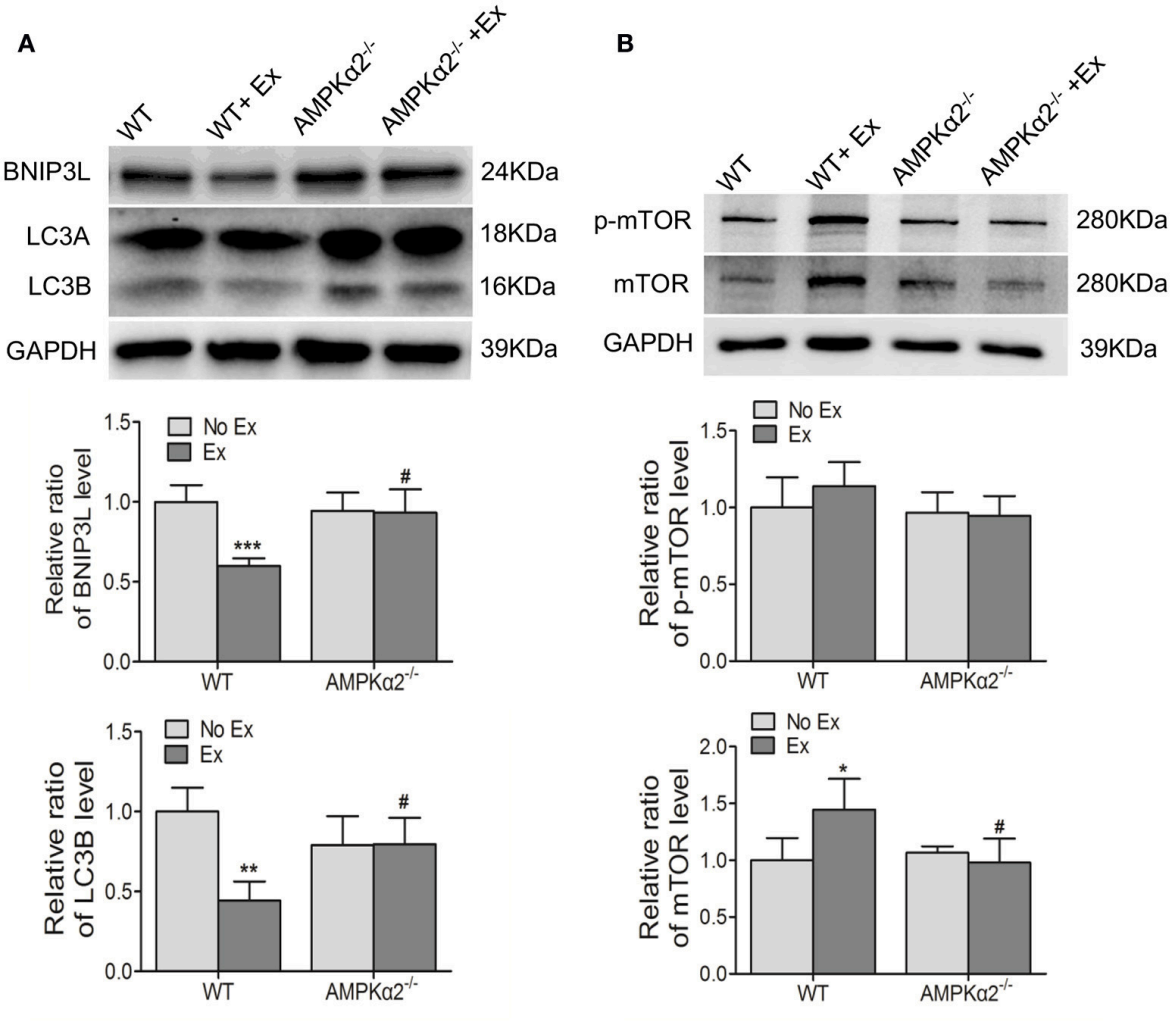
**The changes of mitochondria autophagy-related protein LC3B, BNIP3L, and mTOR during exercise training were dependent on AMPKα2. (A)** Representative western blot images and summarized data showing the expression of LC3B and BNIP3L of aorta from WT and AMPKα2 knockout mice with or without exercise. **(B)** Representative western blot images and summarized data showing the expression of mTOR and p-mTOR of aorta from WT and AMPKα2 knockout mice with or without exercise. The protein levels of LC3B, BNIP3L, p-Mtor, and mTOR were normalized to GAPDH. WT, wild type; Ex, exercise. Values are mean ± SD (*n* = 6 in each group). ^*^p < 0.05; ^**^p < 0.01;^***^p < 0.001 versus WT. ^#^*p* < 0.05 versus WT+Ex.

It has been reported that mitochondrial quantity and quality were controlled by biogenesis, fusion-fission, and autophagy. We therefore examined the effect of exercise on the expression of these relative proteins and the possible role of AMPKα2^−/−^. We found that the expression of LC3B, an indicator of autophagy, and BNIP3L, a mitochondria-associated protein, were decreased in WT mice with exercise compared with WT mice without exercise (Figure 4B). Then we detected the protein expression of mTOR, the major autophagy negative regulator, and its phosphorylation at Ser2448. It was shown that WT exercise mice showed increased mTOR protein content compared to WT mice without exercise, but no significant alteration in phosphorylation activity (Figure 4B). In contrast, there was no significant difference in either LC3B/BNIP3L or mTOR protein levels in AMPKα2^−/−^ mice with exercise compared with these knockout mice without exercise. These results indicated that decreased autophagy may be responsible for exercise-related increase of mitochondrial content, and this effect was dependent on AMPKα2.

In contrast, there was no significant difference of PGC-1α (peroxisome proliferator-activated receptor gamma coactivator 1 alpha) protein expression, the main regulator of mitochondrial biogenesis, in the WT and AMPKα2^−/−^ mice with exercise compared with matched strain without exercise (Figure S2). Similarly, the protein levels of Drp1 (dynamin related protein 1) and Mfn1 (mitofusin 1), fission and fusion markers, also remained unchanged in the WT and AMPKα2^−/−^ mice with exercise compared with matched stains without exercise. (Figure S2). These results indicated that mitochondrial biogenesis, fission and fusion might not be involved in exercise-related mitochondria content increase in the aorta.

### AMPKα2 deficiency attenuates exercise-mediated increase in aortic mitochondrial antioxidant capacity

**Figure 5:**
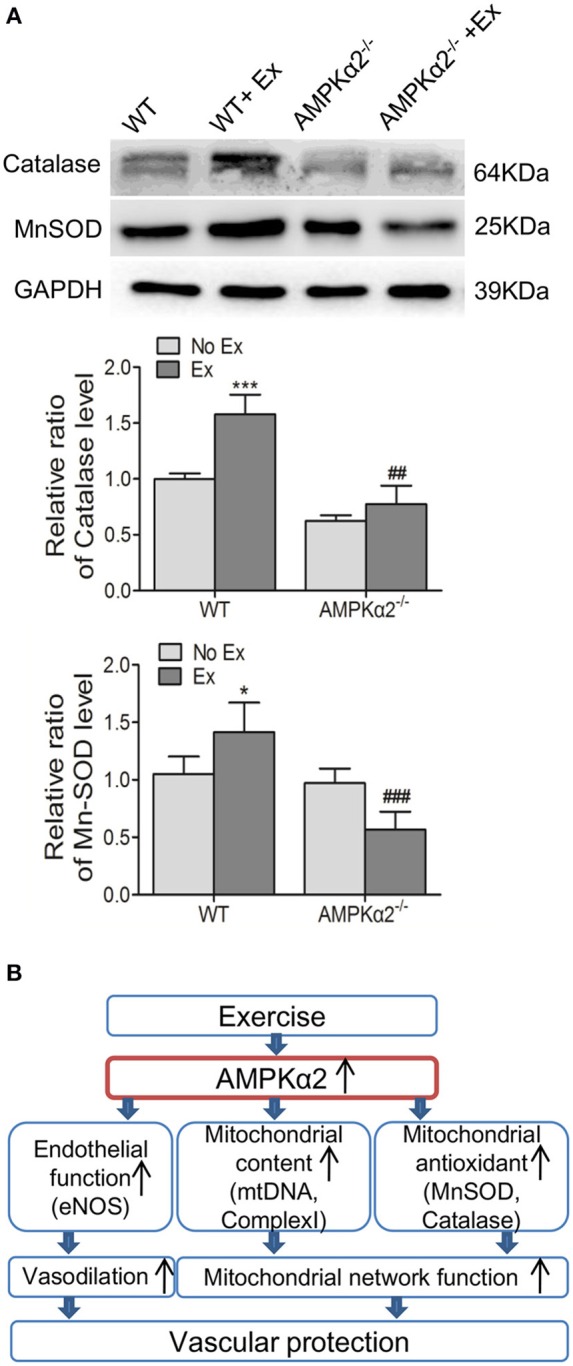
**Exercise training enhanced aortic mitochondrial MnSOD and catalase via AMPKα2. (A)** Representative western blot images and summarized data showing the expression of MnSOD and catalase of aorta from WT and AMPKα2 knockout mice with or without exercise. **(B)** Scheme illustrates that AMPKα2 plays a critical role in exercise-related vascular protection via increasing endothelial and mitochondrial function in the artery. AMPKα2 is activated after chronic exercise training and in turn, mediates increased eNOS expression and activation, increased mitochondrial content (mtDNA and Complex I) and mitochondrial antioxidant capacity (MnSOD and catalase). These events contribute to the promoted vasodilation and mitochondrial network function, resulting in improved vascular function. The protein levels of MnSOD and catalase were normalized to GAPDH. WT, wild type; Ex, exercise. Values are mean ± SD (*n* = 6 in each group). ^*^p < 0.05; ^***^p < 0.001 versus WT. ^*##*^*p* < 0.01; ^*###*^*p* < 0.001 versus WT+Ex.

Finally, we evaluated the effect of exercise on mitochondrial antioxidant capacity and the possible involvement of AMPKα2. MnSOD and catalase are both critical to mitochondrial specific antioxidant defense. Figure 5A shows that the expression of catalase protein was significantly increased in the WT mice by 58% following exercise exposure, but not in AMPKα2^−/−^ mice. MnSOD protein content was significantly increased in WT mice after exercise intervention. Conversely, a marked decrease of MnSOD was observed in AMPKα2^−/−^ mice with exercise compared with those without exercise (Figure 5A). These results indicated that exercise might increase mitochondrial antioxidant response in the aorta, and this effect was dependent on AMPKα2.

## Discussion

In the present study, we provide strong evidence that exercise promoted vasodilation, increased eNOS expression/^*S*1177^-phosphorylation, and increased mitochondrial content and mitochondrial antioxidant capacity, resulting in increased vascular function. Importantly, these beneficial effects are dependent on the presence of AMPKα2 (Figure 5B).

Previous studies show that acute and long-term exercise training potently stimulate AMPK activity in skeletal muscles (Richter and Ruderman, 2009). Goirand et al. and Musi et al. have provided evidence that exercise training could activate cardiac and vascular AMPK in mice (Musi et al., 2005; Goirand et al., 2007). Consistent with these studies, the present study shows that exercise training significantly increased AMPKα2 activity in the vasculature. Furthermore, the present study shows that chronic exercise training increased vasodilation, eNOS expression and phosphorylation in aorta from mice, and these effects were lost in AMPKα2 knockout mice. It has been reported in a previous study that the activation of AMPKα1 was associated with increased vasodilatation and eNOS activation in mouse aorta during exercise training (Kröller-Schon et al., 2012). In this present study, we observed that AMPKα2 activation in response to exercise-related protection also occurs in the vasculature. Moreover, acute exercise activated eNOS associated with the presence of AMPK in mouse aorta (Cacicedo et al., 2011), which is consistent with the present study showing that exercise increased eNOS activity through an AMPKα2 dependent manner.

Increasing evidence demonstrated that maintaining mitochondrial content/function and stability is essential to normal vascular systems (Dromparis and Michelakis, 2013; Kröller-Schon et al., 2013), and that exercise can stimulate key stress signals that control mitochondrial content and function in skeletal muscles (Russell et al., 2014). In the current study, we find that chronic exercise training induces an adaptive increase of mitochondrial quantity in the aorta, including the increased mtDNA and Complex I protein content. Further study shows that the expression of autophagy markers LC3B and BNIP3L (Zhu et al., 2013) was decreased, and that the expression of autophagy inhibitor marker mTOR (Kim et al., 2011) was increased in exercise training. These results indicate that autophagy was decreased in exercise and that the decreased autophagy might be responsible for the increased mitochondrial content in exercise. Consistent with the present study, it has been shown that autophagy is decreased in skeletal muscle cells after exercise (Kim et al., 2012). Besides autophagy, mitochondria content can also be regulated by biogenesis, fission, and fusion (Youle and Narendra, 2011; Ding et al., 2013). In our study, mitochondrial biogenesis and mitochondrial fusion and fission may have no effect on the changes in aorta with exercise training, since the expression of PGC-1α, Mfn1, and Drp1 was not changed in exercised mice compared mice without exercise. In contrast, previous studies suggest that AMPK works through PGC-1α to promote mitochondrial biogenesis in acute exercise in skeletal muscles (Kahn et al., 2005; Reznick and Shulman, 2006), and that endurance training increases Mfn1 content to induce mitochondrial fusion in rat liver (Gonçalves et al., 2016). These results suggest that mitochondrial content may be regulated through different signaling pathways in different cells in exercise training.

Furthermore, our study demonstrates that chronic exercise training upregulated the protein expression of MnSOD and catalase, and the increases are depended on the AMPKα2 isoform. Indeed, it has been reported that antioxidant enzymes were activated by exercise training in rat brains (Marosi et al., 2012; Marcelino et al., 2013), and chronic aerobic exercise training increased aortic mitochondrial antioxidant enzyme in aged rats (Gu et al., 2014). Consistent with our results, AMPKα2 has been reported to be involved in the protective effect of swimming training against isoproterenol-induced ROS production and promote the expression of antioxidant enzymes in mouse hearts (Ma et al., 2015). AMPK activity has been reported to be associated with the redox reaction in different tissues in the cardiovascular system (Ma et al., 2015). Activation of AMPK by 5-aminoimidazole-4-carboxamide ribonucleotide (AICAR) could significantly decrease ROS which was induced by palmitic acid in human aortic endothelial cells and increased expression of the antioxidant thioredoxin (Li et al., 2009). In addition, AMPKα2 suppressed NADPH oxidase expression and reduced ROS production in endothelial cells (Wang et al., 2010) as well as induced manganese SOD (Kukidome et al., 2006).

Finally, our results show that exercise-related increases of eNOS expression/phosphorylation, mitochondrial content and MnSOD/catalase expression were lost in AMPKα2 knockout mice. As demonstrated in the present study, the increased vasodilation and eNOS activation during exercise were decreased in AMPKα2 deficient mice. This may be caused by the finding that AMPK is able to phosphorylate eNOS, thereby leading to eNOS activation and increased NO production (Morrow et al., 2003), and that the loss of AMPKα2 may prevent exercise-related eNOS activation. As for the increased expression of eNOS, mitochondria content and increased expression of MnSOD and catalase in aorta with exercise, the increased AMPKα2 may induce the expression of these proteins through direct interaction with the nucleus in WT mice as previously reported (McGee et al., 2003; Jørgensen et al., 2006). In AMPKα2 knockout mice, however, these corresponding proteins were not increased due to the lack of AMPKα2 to promote the expression of these proteins. Indeed, a recent report showed that AMPK signaling is required for the metabolic response to exercise *in vivo*, and AMPK activation was proposed as a regulatory mechanism that underlies exercise-induced glucose uptake in muscles, thereby leading to increased systemic insulin sensitivity (Steinberg and Jørgensen, 2007).

It has been shown that AMPK plays a protective role in diabetes and hypertension. For example, Wang et al. reported that AMPK activation was reduced in diabetic mice, and that metformin, an AMPK activator, normalized the acetylcholine-induced endothelial relaxation (Wang et al., 2009). Sun et al. found that resveratrol, a compound that activates AMPK, induced vasodilation and lowered blood pressure in DOCA-hypertensive mice(Sun et al., 2015). Metformin attenuated cytokine-induced expression of proinflammatory factors via AMPK activation in human umbilical vein endothelial cells (Hattori et al., 2006). The present finding that exercise improved aortic endothelial and mitochondrial function via AMPKα2 activation suggests that AMPKα2 may play a critical role in exercise-related improvement of vascular function in diabetes and hypertension.

In summary, our study shows that chronic exercise training mediates vascular protection through improving aortic endothelial and mitochondrial function, and that vascular AMPK isoform AMPKα2 is a key signaling molecule that mediates the protective effects of exercise in the vasculature. These findings may provide a novel mechanism in exercise-related cardiovascular protection.

## Author contributions

XC designed the study, performed the experiments, collected and analyzed the data, and wrote and revised the final version of the manuscript. XA and DC contributed to data collection. WS, YZ, and PG designed the study. MY, WH, and PG critically revised the final version of the manuscript. All authors read and approved the final version of the manuscript.

## Funding

This study was supported by the National Natural Science Foundation of China (81100184, 81230071, 81300089, 81200203, 91539202, and 81570221), the Scientific Fund of Shanghai Jiao Tong University School of Medicine (14XJ10042), the Pujiang Program of the Shanghai Science and Technology Committee (14PJ1406400), the Scientific Research Foundation for the Returned Overseas Chinese Scholars of the State Education Ministry, and the Shanghai Medical Bureau Fund (201540037).

### Conflict of interest statement

The authors declare that the research was conducted in the absence of any commercial or financial relationships that could be construed as a potential conflict of interest.
