# Supplementary material for: Chronic Exercise Training Improved Aortic Endothelial and Mitochondrial Function via an AMPKα2-Dependent Manner
Source: Front Physiol. 2016 Dec 21;7:631. doi: 10.3389/fphys.2016.00631 (PMC5175474; doi:10.3389/fphys.2016.00631)
Supplement: Supplementary file 1 [file Presentation1.PDF]

Table 1

Body weights, heart rate, systolic blood pressure and diastolic blood pressure of four groups mice.

	WT	WT+Ex	AMPKα2$^{-/-}$	AMPKα2$^{-/-}$+Ex
Body Weight (g)	30.9 ± 3.4	32.3 ± 2.5	28.7 ± 39	29.1 ± 2.2
Heart Rate (times/min)	661.0 ± 91.9	544.2 ± 22.1*	652.5 ± 39.3	582.6± 43.8*
Systolic Blood Pressure (mmHg)	119.8 ± 7.4	111.7 ± 6.0	119.8 ± 4.2	120.8 ± 5.7
Diastolic Blood Pressure (mmHg)	75.3 ± 6.1	68.0 ± 3.3*	72.5 ± 9.3	68.9 ± 10.8

WT, wild type; Ex, exercise. Value are mean ± SEM (n = 6 in each group)
*P < 0.05 versus WT mice.

Fig 1

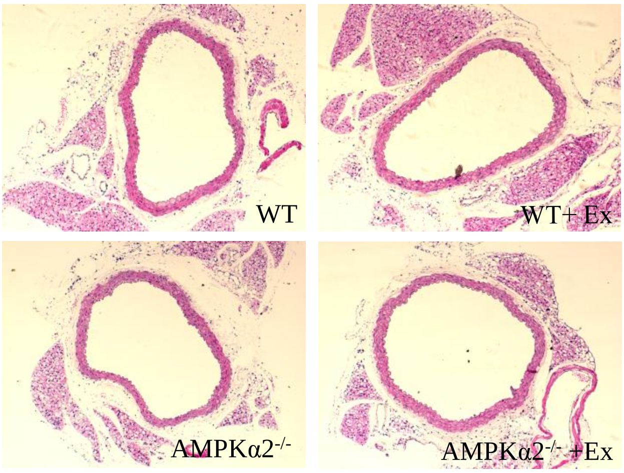

Representative micrographs of hematoxylin&eosin (H&E)-stained sections of aorta from WT and AMPKα2 knockout mice with or without exercise. WT, wild type; Ex, exercise.

Fig 2

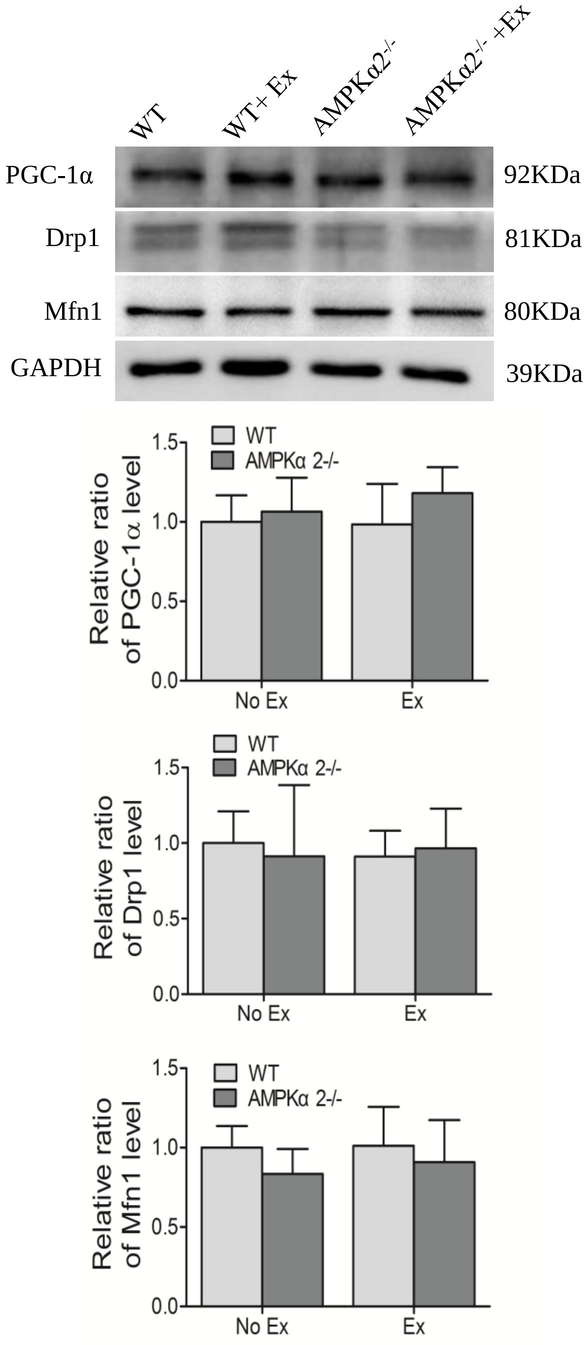

Representative western blot images and summarized data showing the expression of PGC-1α, Drp1 and Mfn1 of aorta from WT and AMPKα2 knockout mice with or without exercise. The protein levels of PGC-1α, Drp1 and Mfn1 were normalized to GAPDH. WT, wild type; Ex, exercise. Value are mean ± SEM (n = 6 in each group).
