# Supplementary material for: Chronic Exercise Training Improved Aortic Endothelial and Mitochondrial Function via an AMPKα2-Dependent Manner
Source: Front Physiol. 2016 Dec 21;7:631. doi: 10.3389/fphys.2016.00631 (PMC5175474; doi:10.3389/fphys.2016.00631)
Supplement: Supplementary file 2 [file Presentation2.PDF]

Figure R1

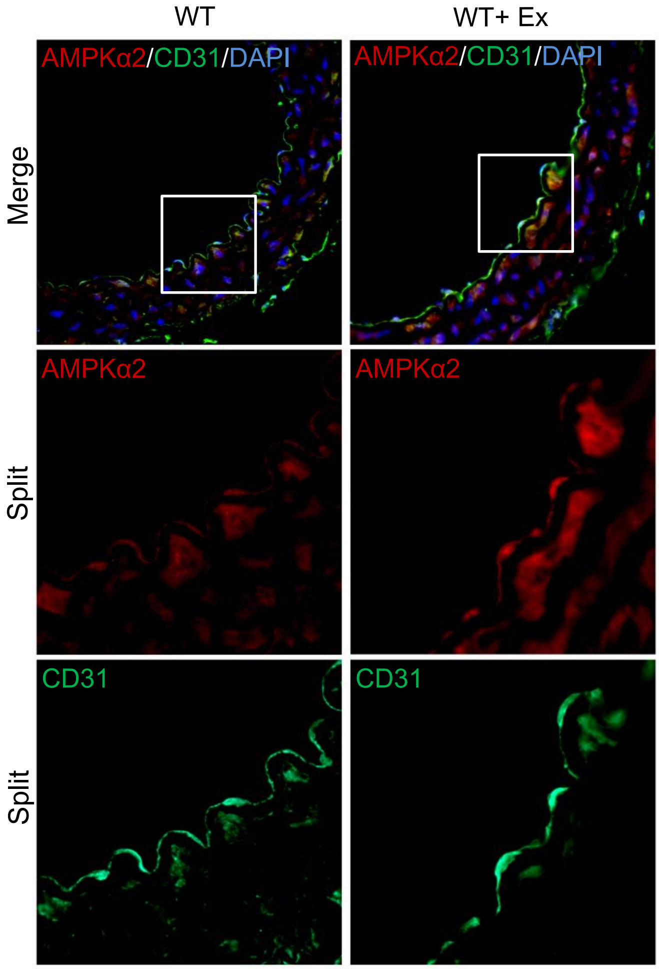

Representative immunofluorescence images showing the expression of AMPKα2 and CD31 in aorta, in which AMPKα2 fluorescence-positive cells were stained in red, CD31 fluorescence-positive cells were stained in green, and nuclei were counterstained with DAPI (blue).

Figure R2

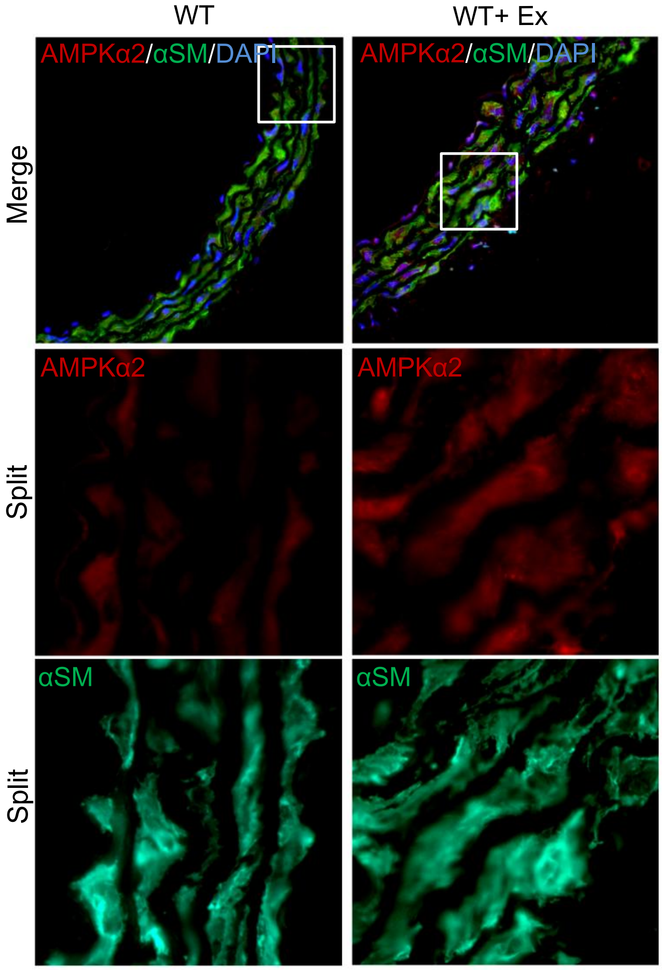

Representative immunofluorescence images showing the expression of AMPKα2 and αSM in aorta, in which AMPKα2 fluorescence-positive cells were stained in red, αSM fluorescence-positive cells were stained in green, and nuclei were counterstained with DAPI (blue).

Figure R3

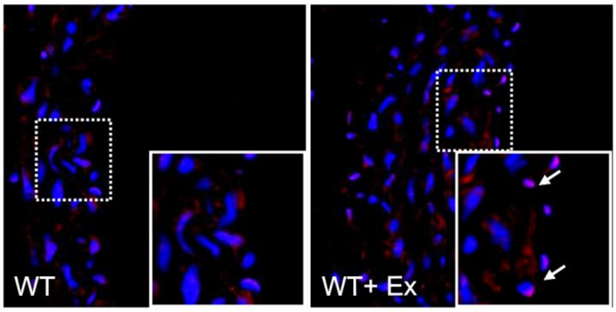

Representative immunofluorescence images showing the expression of AMPKα1 in aorta, in which fluorescence-positive cells were stained in red (arrow) and nuclei were counterstained with DAPI (blue).

Figure R4

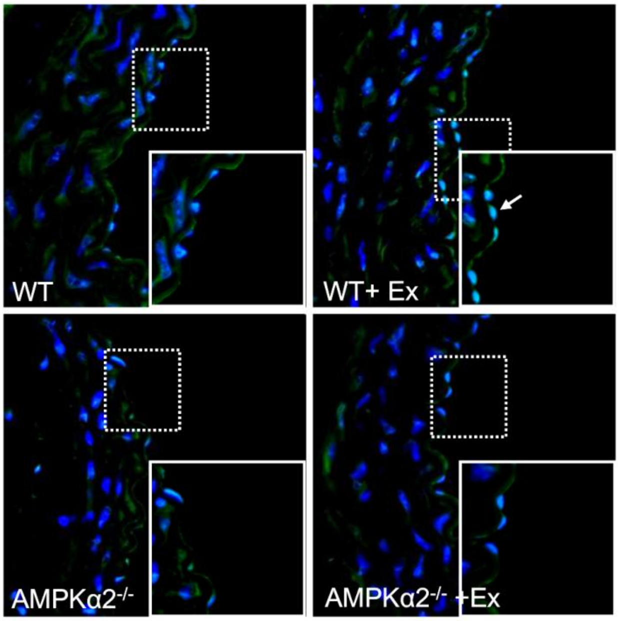

Representative immunofluorescence images showing the expression of eNOS in aorta, in which fluorescence-positive cells were stained in green (arrow) and nuclei were counterstained with DAPI (blue).
