# Supplementary material for: Chronic Exercise Training Improved Aortic Endothelial and Mitochondrial Function via an AMPKα2-Dependent Manner
Source: Front Physiol. 2016 Dec 21;7:631. doi: 10.3389/fphys.2016.00631 (PMC5175474; doi:10.3389/fphys.2016.00631)
Supplement: Supplementary file 3 [file Presentation3.PDF]

Figure R5

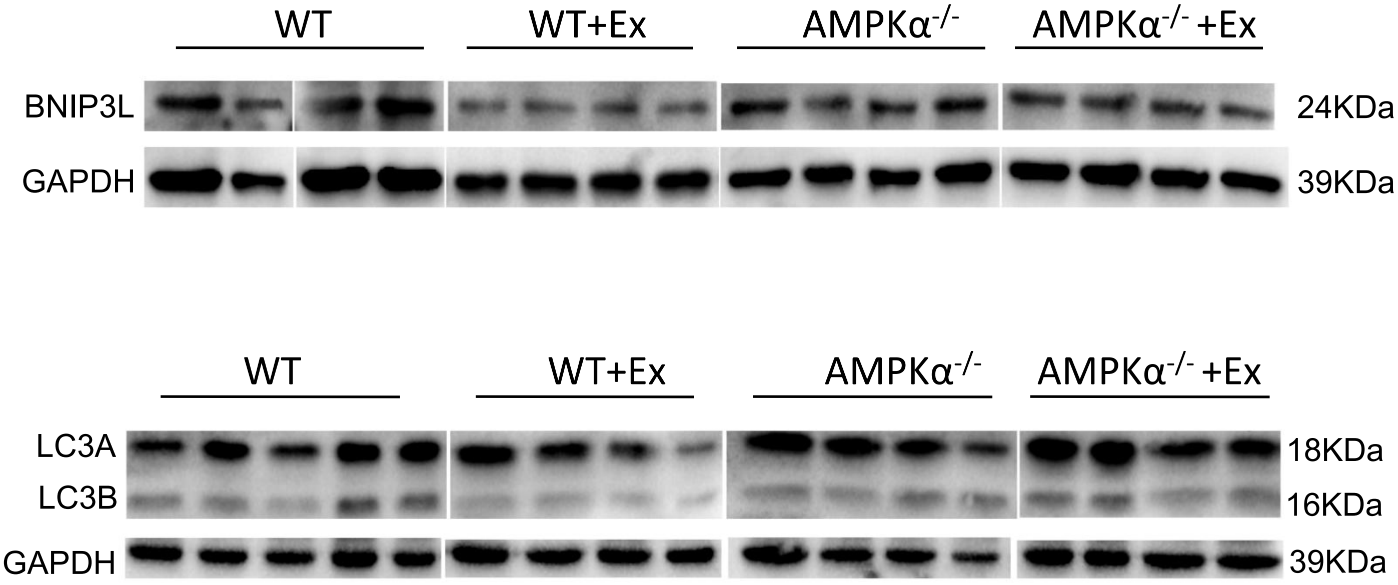

The original western blot images of LC3B and BNIP3L of aorta from WT and AMPKα2 knockout mice with or without exercise.
